# Characteristics of crude oil‐degrading bacteria *Gordonia iterans* isolated from marine coastal in Taean sediment

**DOI:** 10.1002/mbo3.754

**Published:** 2018-10-19

**Authors:** Hyun‐Sook Kim, Ke Dong, Jinsoo Kim, Sang‐Seob Lee

**Affiliations:** ^1^ Department of Biological Engineering Kyonggi University Suwon‐si Korea; ^2^ Department of Life Science Kyonggi University Suwon‐si Korea

**Keywords:** *alk*B gene, *Gordonia iterans* Co17, marine and costal ecosystem, petroleum contamination biodegradation, whole genome de novo sequencing

## Abstract

Crude oil is a major pollutant of marine and coastal ecosystems, and it causes environmental problems more seriously. It is believed ultimate and complete degradation is accomplished mainly by microorganisms. In this study, we aim to search out for bacterial strains with high ability in degrading crude oil. From sediments contaminated by the petroleum spilled in 2007, an accident in Taean, South Korea, we isolated thirty‐one bacterial strains in total with potential application in crude oil contamination remediation. In terms of removal percentage after 7 days, one of the strains, Co17, showed the highest removal efficiency with 84.2% of crude oil in Bushnell‐Haas media. The Co17 strain even exhibited outstanding ability removing crude oil at a high salt concentration. Through the whole genome sequencing annotation results, many genes related with *n*‐alkane degradation in the genome of *Gordonia* sp. Co17, revealed alkane‐1‐monooxygenase, alcohol dehydrogenase, and Baeyer–Villiger monooxygenase. Specially, for confirmation of gene‐level, *alk*B gene encoding alkane hydroxylase (alkane‐1‐monooxygenase) was found in the strain Co17. The expression of *alk*B upregulated 125‐fold after 18 hr accompany with the removal of *n*‐alkanes of 48.9%. We therefore propose the strain *Gordonia iterans* Co17, isolated from crude oil‐contaminated marine sediment, could be used to offer a new strategy for bioremediation with high efficiency.

## 1 INTRODUCTION

Acute petroleum pollution in the soil is usually caused by spilling or leakage of oil storage tanks. Meanwhile in the ocean, it occurs in places such as pipeline terminals or oil refineries. Oil spill accident happens time to time, take the well‐known ones for example, Amoco Cadiz in France in 1978, Exxon Valdez in 1989, and the Prestige in 2002 (ITOPF, 2006). In South Korea, crude oil spills occurred along the Sea Prince in 1995 and the Hebei Spirit in 2007 resulting in leakage of about 5,000 tons and 10,500 tons at each location, respectively. Petroleum that has been leaked by accident may take a long time to recover.

All the petroleum contaminants will eventually leak into the marine ecosystem, and will be degraded naturally by physicochemical and biological activity. Natural degradation takes a long time and is caused by evaporation, oxidation, spreading, dispersion, and waves (Hazen, Prince, & Mahmoudi, 2016; McGenity, Folwell, McKew, & Sanni, 2012).Emulsification and biodegradation are triggered by oil‐degrading microorganisms. Sinking or sedimentation is the most dangerous phenomenon induced by oil seepage into the sea and changes under anaerobic conditions. These chronic oil pollutants usually persist in the ocean over a long period of time and seriously affect the ecosystem. Petroleum contaminates habitats of marine organisms such as oceanic fish and seaweed. The oil pollution affects humans as well. Crude oil spills directly affect the lungs and indirectly affect the whole body following consumption of food derived from marine habitats (Gardner, Yevich, Harshbarger, & Malcolm, 2011; Orisakwe, Akumka, Njan, & Afonne, 2004) .

Marine oil pollution is reported to be biodegraded by bacteria, algae, and fungi. To date, oil‐degrading marine bacteria were reported such as genera *Alcanivorax* (Hara, Syutsubo, & Harayama, 2003; Yakimov, Golyshin, Lang, & Moore, 1998), *Cycloclasticus* (Kasai, Kishira, & Harayama, 2002), *Marinobacter* (Gerdes, Brinkmeyer, Dieckmann, & Helmke, 2005)*, Oceanobacter* (Teramoto, Suzuki, Okazaki, & Hatmanti, 2009), *Oleibacter* (Teramoto, Ohuchi, Hatmanti, & Darmayati, 2011), *Oleispira* (Coulon, McKew, Osborn, & McGenity, 2007; Kube, Chernikova, Al‐Ramahi, & Beloqui, 2013), *Oleiphilus* (Golyshin, Chernikova, Abraham, & Lünsdorf, 2002),* Shewanella* (Gerdes et al., 2005), and *Thalassolituus* (Yakimov, Giuliano, Denaro, & Crisafi, 2004). These bacteria exhibit oil degradation under aerobic conditions using monooxygenases such as alkane hydroxylase. The alkanes are degraded via terminal or sub‐terminal oxidation. The alkane hydroxylase (*alk*B) catalyzes the terminal oxidation of medium‐ and long‐chain alkanes, and converts alkane to alkanols (Parthipan, Preetham, Machuca, & Rahman, 2017; Rojo, 2009). Additionally, Baeyer–Villiger monooxygenase (*alm*A) is related sub‐terminal oxidation of C_20_‐C_32_ alkane (Liu, Galzerani, Mbadinga, & Zaramela, 2018; Minerdi, Zgrablic, Sadeghi, & Gilardi, 2012) and The flavin‐dependent monooxygenase (*has*) degraded *n*‐alkanes to fatty acids (Wang & Shao, 2012).

In this study, we have searched for crude oil high efficiency degrading strains, bacteria *Gordonia* sp. Co17 was isolated from the oil‐contaminated site Hebei Spirit in year of 2007, and the environmental factor for bioremediation efficiency was determined. Through the whole genome de novo sequencing, the genes of *Gordonia* were annotated. Especially with the *alk*B gene, which is a key enzyme in the alkane degradation pathway identified in oil‐degrading bacteria, we constructed the phylogenetic tree with a closely associated *alk*B gene family member including bacteria. In addition, the degradation rate was measured and the amount of *alk*B gene expression was examined by quantitative real‐time PCR (qRT‐PCR).

## 2 MATERIALS AND METHODS

### 2.1 Bacteria isolation and identification

The sample was collected from the oil spill area in Taean, South Korea (36°47′16″N 126°08′37″E) in March 2008. This area was contaminated by crude oil in 2007 because of oil spill accident by Hebei Sprit. Sea water samples were collected from depths of 5 m from surface using sterile water sample bottle. And sediment samples were collected using a sterilized grab along the coastal then sealed in to a plastic aseptic bag. Samples were stored in icebox and transported to the laboratory within 1 day after being collected.

Sea water was filtered using a membrane filter (0.2 μm pore size), and the obtained filtered membrane was cultured in 50 ml of Bushnell Hass media (BH media, 0.2 g/L MgSO_4_, 0.02 g/L CaCl_2_, 1.0 g/L KH_2_PO_4_, 1.0 g/L K_2_HPO_4_, 1.0 g/L KNO_3_, 0.05 g/L FeCl_3_ and the pH was adjusted to 7.0 ± 0.2 (Atlas, 2014)). The sediment sample of 1 g weight was cultured the same way. Two percent NaCl within BH media and crude oil were added to the media and incubated at 25°C for 1 week. After obtaining the enriched culture, the suspension was diluted to 10^−6^ and spread onto 1.5% agar of BH media, incubated at 25°C.

All isolated strains were conducted of screening of crude oil degradation activity in triplet, and the selected strain was identified using the universal bacterial primers. The pairwise 16S rRNA gene sequence similarity was determined using the Ezbiocloud server (Yoon, Ha, Kwon, & Lim, 2017).

### 2.2 Detection of crude oil degradation efficiency of strains

Kuwait crude oil was used to conduct bioremediation screening of petroleum. The crude oil (0.1%, v/v) was injected into BH media, and 1.0 g/L (w/v) of single bacteria was inoculated. The bacteria harvested at exponential phase, was washed out with 1X PBS buffer, and then the wet weight was measured after inoculated and cultured at pH 7.0, 25°C for 7 days. After cultivation, the residual oil contaminant was extracted with dimethyl chloride. Dimethyl chloride was injected in half volumes and shaken for 30 min. The aqua‐phase was replaced with anhydrous sodium sulfate to remove water in organic‐phase. Dimethyl chloride was evaporated, and the residual oil contaminant was redissolved in 2 ml of dimethyl chloride.

The extracted oil contaminant was examined by GC‐FID (Gas Chromatography‐Flame Ionization Detector) and GC‐MS (Gas Chromatography‐Mass Spectrometry). The GC‐FID column used a capillary DB‐1HT, oven condition was maintained at 40°C for 3 min, increased by 12°C/min to 275°C, and held for 12 min. The injector temperature was 280°C, and the detector temperature was 340°C. Nitrogen gas was used as the carrier gas. The GC‐MS analysis was conducted under the following conditions: oven temperature 40°C for 4 min; increased by 5°C/min to 320°C; held for 15 min; and helium gas was used as the carrier gas. The mass spectrum was recorded from *m*/*z* 50 to 720 amu, in the total ion chromatography (TIC) mode. The C_8_–C_40_ alkane calibration standard (SUPELCO) and total petroleum hydrocarbon (TPH) mixture 1 (SUPELCO, C_10_–C_28_ alkane mixture) were used as standard materials.

To calculate the crude oil removal efficiency (*R*_E_), the peak area was used, excluding the Unresolved Complex Mixture (UCM). The *R*_E_ was calculated according to the modified Equation (1) (Deng, Li, Liang, & Yi, 2014; Zhang, Gai, Hou, & Yang, 2010).

(1) $$R_{E}\;=\;\frac{C_{0}-C_{t}}{C_{0}}\;\times \;100\left(\%\right)$$

*R*_E_, *C*_0_, and *C*_t_ denote removal efficiency, control (bacteria no treatment) concentration of crude oil, and the remaining crude oil concentration following bacterial treatment, respectively.

### 2.3 Effect of environmental variables on crude oil removal efficiency

We investigated crude oil degradation efficiency according to environmental conditions such as temperature and NaCl tolerant during 3 days, the residual oil was extracted and *R*_E_ value was calculated. The temperature conditions were 10, 20, 25, 37, and 40°C, and the NaCl concentration were 0%, 2%, 4%, 6%, 8%, 10%, and 12% (w/v) in BH media. Excluding the factors underlying the changes, the conditions were maintained at 25°C, 2% of NaCl (w/v), 1.0 g/L (w/v) of bacteria, 0.1% crude oil, and pH 7.0.

### 2.4 Whole genome de novo sequencing and analysis

Whole genome sequencing and analysis were conducted in Macrogen. Genomic DNA sequencing was performed on the PacBio RS II platform and Illumina HiSeq platform. By mapping the HiSeq reads to first assembled genome sequence, we can see the mapping result that shows a slight difference from the assembly result. Also, we can get a consensus sequence with higher quality through the self‐mapping step. After whole genome or draft genome is assembled, the location of protein‐coding sequence, tRNA genes, and rRNA genes were analyzed by ARAGORN v1.2 (Laslett & Canback, 2004) and RNAmmer (v1.2) (Lagesen, Hallin, Rødland, & Stærfeldt, 2007), respectively. Then, their functions are annotated. PacBio sequencing reads were de novo assembled with hierarchical genome assembly process (HGAP, v3.0) and annotation was by Prokka (v1.12b) (Seemann, 2014).

### 2.5 Phylogenetic analysis of *alk*B and gene expression

To distinguish with previously reported *alk*B gene, we compared the *alkB* gene of *Gordonia* sp. Co17 with the *alkB* gene sequences obtained from NCBI (Supporting Information Table S1). The *alkB* gene of *Gordonia* sp. Co17 was sequenced by Macrogen (South Korea) against the PCR product amplified using primers alkBF (5′‐ATCAAYRCVGCVCAYGARYTVGGBCACAAG‐3′) and alkBR (5′‐SGGRTTCGCRTGRTGRTCRCTGTGNSGYTG‐3′) (Shen, Young, Hsieh, & Lin, 2010).

The resulted *alk*B sequence of *Gordonia* and other sequences were assembled using SeqMan software (DNASTAR Inc.). The phylogenetic analysis was constructed using the MUSCLE program in MEGA 6, and pairwise distances were calculated with the Kimura 2‐parameter model, which was also used to determine the confidence levels of the branches (Felsenstein, 2009; Kimura, 1989; Tamura, Stecher, Peterson, & Filipski, 2013). The analyzed sequence was submitted to GeneBank with accession number of KY312029. The *alk*B gene (EU853422) of *Alcanivorax dieselolei* II‐D‐3 was outside the group.

Total RNA was extracted according to the manual provided by the Hybrid‐R^TM^ Blood RNA kit (GeneAll). To synthesize cDNA, TransScript First‐Strand cDNA Synthesis Super Mix kit and 1 µl (0.1 µg/µl random primer were used. Real‐time PCR (CFX 96, Bio‐Rad) was performed with 10 µl 2X iQ^TM^ SYBR green mixture (Bio‐Rad), 50 ng/µl cDNA, and each 1 µl of 10 pmol primer pairs RT_*alk*BF and RT_*alk*BR (5′‐CTGCGATCTTCGGCTGGGA‐3′/5′‐GCCAGGTAGTTCACGGTCT‐3′, in this study), and filled up to 20 µl of DEPC. The expression of *alk*B gene was normalized with 16 s rRNA gene.

## 3 RESULTS

### 3.1 Bacterial isolates and the strains with crude oil removal ability

In total, 144 strains were isolated from the sea water and sediment samples contaminated by crude oil (Supporting Information Table S2) and 31 strains among these exhibited outstanding ability in degrading crude oil with the degradation efficiency more than 65% (Table 1) on 7 days. Among 31 strains, 20 strains were identified into *Gordonia*,* Microbacterium*, and *Rhodococcus* genus from *Actinobacteria* phyla, 2 strains were identified *Bacillus* genus from *Firmicutes* phyla. And 9 strains were identified into *Albirhodobacter*,* Ochrobactrum*,* Pseudomona*,* Rhizobium*,* Shewanella*,* and Vibrio* genus from *Proteobacteria* phyla.

**Table 1:** Identification of 31 strains exhibited ability in degrading crude oil

Identification				Efficiency (%)
Phylum	Genus	Species	Strain	
*Actinobacteria*	*Gordonia*	*iterans*	Co17	84.23
		*malaquae*	LNB035‐2	69.23
			F3	69.15
		*alkanivorans*	9004‐010	69.20
			9004‐035	67.11
		*polyisoprenivorans*	F8	69.15
			LNB024‐2	68.54
	*Rhodococcus*	*wratislaviensis*	Py 2‐4	80.87
		*pyridinivorans*	B‐0‐12	76.59
		*equi*	PHEN12	69.75
			20D‐30‐2	67.89
			20D‐30‐4	66.80
		*jialingiae*	20S‐25‐12	68.87
			20S‐25‐5	68.74
			20S‐25‐11	68.65
			20S‐25‐10	68.44
			20S‐25‐4	66.75
			20S‐25‐3	65.77
		*qingshengii*	Wonp9	67.15
	*Microbacterium*	*esteraromaticum*	20S‐25‐13	65.43
*Firmicutes*	*Bacillus*	*anthracis*	TS13	69.00
		*vietnamensis*	UL6	67.75
*Proteobacteria*	*Shewanella*	*aquimarina*	Po7	75.88
		*haliotis*	Po6	69.21
	*Ochrobactrum*	*anthropi*	S. PHEN6	72.31
			PHEN13	69.10
	*Pseudomonas*	*migulae*	Wonp3	69.80
		*mendocina*	UL13	69.15
	*Albirhodobacter*	*marinus*	20S‐25‐14	68.32
			20S‐25‐1	66.12
	*Rhizobium*	*halophytocola*	20S‐25‐8	65.87

**Figure 1:**
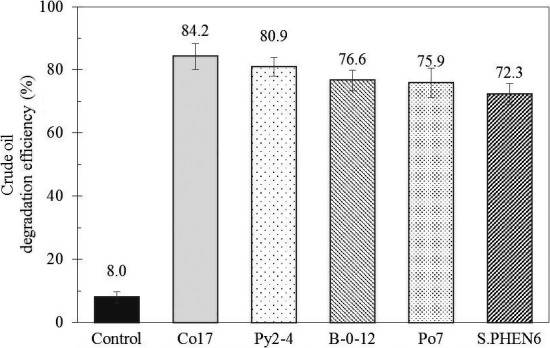
Crude oil degradation efficiency obtained through GC‐FID, GC‐MS. Each error bar represents *SD* of three independent experiment and triplet. Crude oil degradation ability was examined under 25°C, 2% of NaCl, inoculated with 1.0 g/L of bacteria, 0.1% of crude oil, and pH 7.0, at 150 rpm on 7 days

In particular, the strain Co17, Py2‐4, B‐0‐12, Po7, and S. PHEN6 were exhibited *R*_E_ of crude oil 84.2%, 80.9%, 76.6%, 75.9%, and 72.3%, respectively (Figure 1). The initial crude oil concentration was about 1,000 mg crude oil/L, after 7 days, the residual oil was 158.6, 193.0, 236.1, 240.2, and 281.2 mg crude oil/L, respectively. These strains were identified as *Gordonia iterans* IFM 10348^T^ (similarity 99.86%), *Rhodococcus wratislaviensis* N805^T^ (similarity 99.23%),* Rhodococcus pyridinivorans* PDB9^T^ (similarity 99.95%),* Shewanella aquimarina* SW‐120^T^ (similarity 99.39%), and *Ochrobactrum anthropi* ATCC49188^T^ (similarity 99.85%), respectively. Through screening experiments, *Gordonia*, which had the highest crude oil degradation efficiency, was selected as a following experimental strain. The strain *Gordonia itrans* Co17 was gram‐positive, rod shaped (0.2–0.3 × 0.4–0.6 μm) aerobic, non‐motile and shown 0%–9% (w/v) NaCl tolerant (Kang, Ming, Gonoi, & Chen, 2014). To date, it was reported that the *Gordonia* species were isolated from the soil (Borzenkov, Milekhina, Gotoeva, & Rozanova, 2006; Hong, Kim, & Cho, 2010; Nicdao & Rivera, 2012; Saeki, Sasaki, Komatsu, & Miura, 2009), while our results provide meaningful evidence that *Gordonia* species in our study was obtained from marine sample.

### 3.2 Crude oil degradation ability of *Gordonia* sp. Co17

Through the GC‐FID and GC‐MS analysis data, the strain *G. iterans* Co17 exhibited degradation activity (Table 2 and Supporting Information Figure S1). The *n*‐alkanes from octane (C_8_) to octadecane (C_32_) showed degradation averagely 92.1%, meanwhile the hentriacontane (C_31_) showed only 19.0%. In the case of aromatic compounds, naphthalene and anthracene showed degraded 55.3% and 63.2%, respectively. In particular, hopane, known as molecular fossil, is reported to be difficult to purify biologically, and is present in crude oil in the form of 28‐Nor‐17.alpha. (H) –hopane. After 7 days of application of *G. iterans* Co17, 28‐Nor‐17.alpha. (H) ‐hopane changed to 29‐Nor‐ (17.alpha.H, 21.beta.H) ‐hopane and the efficiency was 28.9%.

**Table 2:** Degradation of alkanes (C_8_–C_32_) by *Gordonia* sp. Co17 in the BH media after 7 days at 28°C, 150 rpm

*n*‐alkane	Degradation efficiency (%)
Octane C_8_	92.7
Nonane C_9_	98.8
Decane C_10_	95.0
Undecane C_11_	95.7
Dodecane C_12_	97.7
Tridecane C_13_	99.3
Tetradecane C_14_	95.2
Pentadecane C_15_	93.3
Hexadecane C_16_	97.4
Heptadecane C_17_	96.7
Octadecane C_18_	95.7
Nonadecane C_19_	93.1
Eicosane C_20_	96.1
Henicosane C_21_	98.0
Docosane C_22_	97.7
Tricosane C_23_	96.8
Tetracosane C_24_	98.5
Pentacosane C_25_	99.0
Hexacosane C_26_	97.5
Heptacosane C_27_	98.3
Octacosane C_28_	96.3
Nonacosane C_29_	86.0
Triaconatane C_30_	93.6
Hentriacontane C_31_	19.0
Octadecane C_32_	75.1

### 3.3 Effect of environmental variables on crude oil removal efficiency of strains *Gordonia* sp. Co17

**Figure 2:**
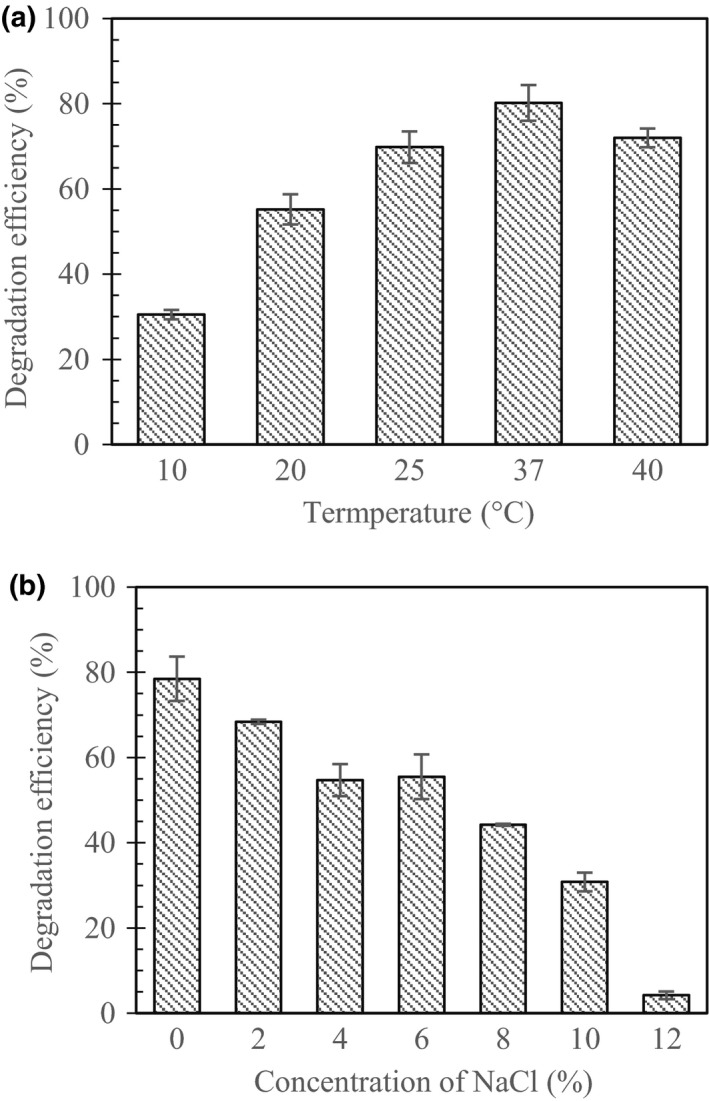
Effects of temperature (a), concentration of NaCl (b) on the degradation of crude oil by strain Co17 on 3 days. Each error bar represents standard deviation of three replicated

At varying temperatures of 10, 20, 25, 37, and 40°C condition, the strain Co17 showed *R*_E_ values of 30.5%, 55.2%, 69.8%, 80.2%, and 72.0%, respectively, (Figure 2a) at 3 days. When cultivated at 37°C, the crude oil degradation ability was the best and the residual oil was 199.2 mg crude oil/L. The results of NaCl concentration (0%, 2%, 4%, 6%, 8%, 10%, and 12% [w/v]) were 78.5%, 68.4%, 54.7%, 55.5%, 44.2%, 30.8%, and 4.2%, respectively, (Figure 2b) at 3 days. As a result of survey according to environmental conditions, it showed the highest *R*_E_ values of 80.2% at 37°C. Meanwhile at low temperature 10°C, cells exhibit 30.5% of *R*_E_. In other condition, the efficiency of salinity was more than 50% *R*_E_ at 0%–6% NaCl concentration; however, the *R*_E_ was remarkably decreased at more than 10% NaCl in the BH media. Although strain Co17 was isolated from the marine habitat, it showed a high *R*_E_ of 78.5% even at without any salinity.

### 3.4 Whole genome de novo sequencing analysis

**Figure 3:**
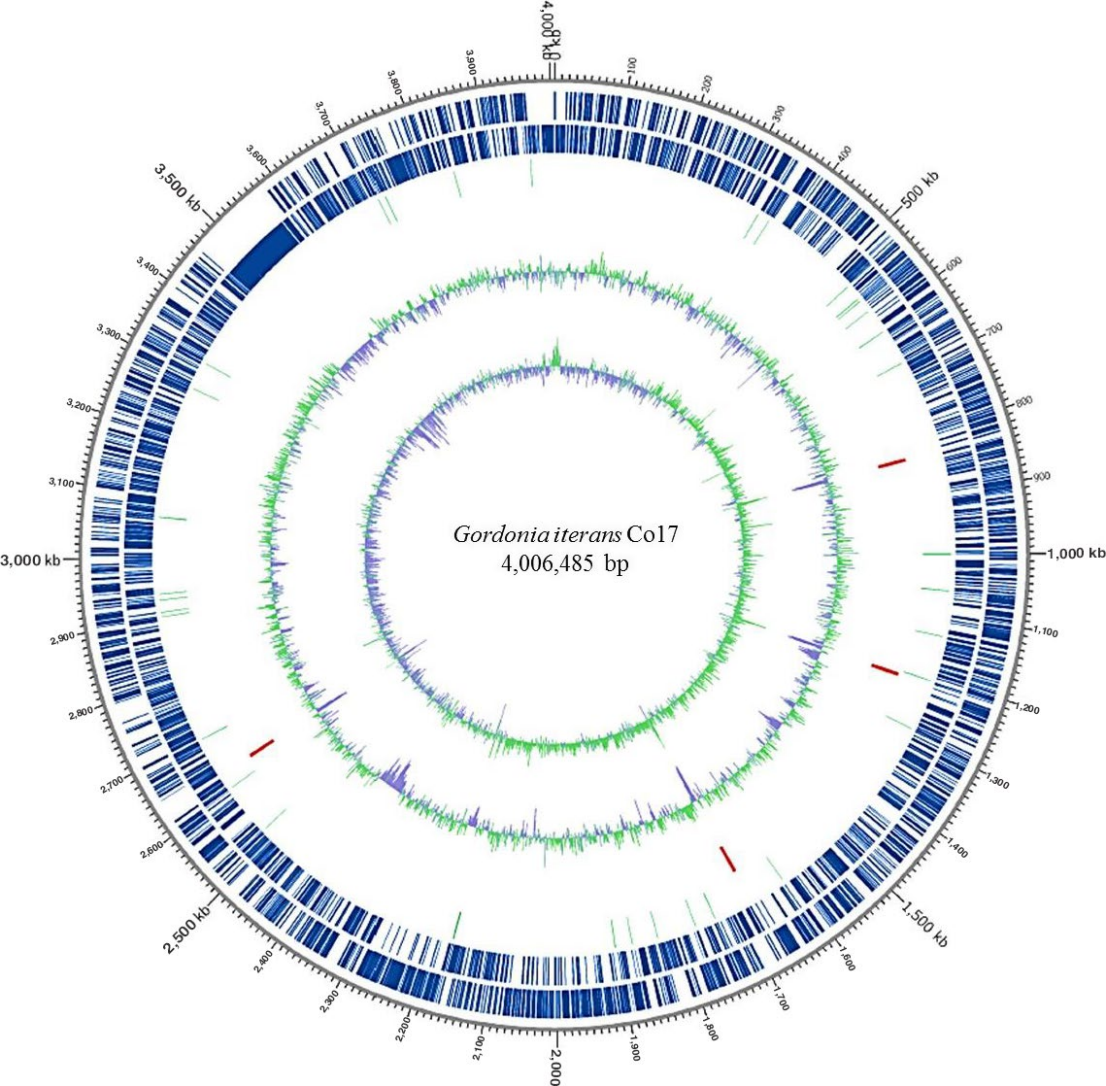
Circular genome map of *Gordonia iterans* Co17. Circular map was drawn by applying the annotated results. Marked characteristics are shown from outside to the center: labeled of genome size, CDSs on forward strand, CDSs on reverse strand, tRNA, rRNA, GC content, and GC skew

The resulting genome assembly sequencing (CP027433) of *Gordonia* sp. Co17 produced one contig with a length of 4,006,485 bp (68.73% GC content), and 3,598 CDS. And genome annotation was conducted 51 of tRNA and 12 rRNA. The general feature of *Gordonia* sp. Co17 was indicated in Supporting Information Table S3 and circular map showed in Figure 3.

Through the annotation results, many genes related with crude oil degradation in the genome of *Gordonia* sp. Co17 were found in the categories of lipid transport and metabolism (I), inorganic transport and metabolism (P), and amino acid transport and metabolism (E) (Table 3). The *Gordonia* sp. Co17 has long‐chain hydrogenase such as *alk*B, *alma* and *has*, and 23 gene of short‐chain hydrogenase such as P450 cytochrome (Fu, Lai, Dong, & Wang, 2018). Briefly, they have two *alk*B gene (C6V83_00720 and C6V83_14510) and four *alm*A (C6V83_07075, C6V83_07450, C6V83_11880, and C6V83_15530). And confirmed three *has* genes and eight genes of alcohol dehydrogenase (*adh*,* adh*T, and *adh*B) were also confirmed in *Gordonia* sp. Co17. Moreover, single *lcf*B gene and three *Fad*D genes encoded long‐chain fatty acid CoA ligase (EC 6.2.1.3) that converted long‐chain fatty acid with a complex structure such as aromatic compounds to acyl‐CoA. As well as the strain Co17 was found to have acetyl‐CoA acetyltransferase, an aromatic compound degrading enzyme, and LysR family transcriptional regulator, a phenolic compound degrading enzyme.

**Table 3:** Number of genes associated with general functional categories

Code	Value	% age	Description
J	156	4.47	Translation, ribosomal structure and biogenesis
A	1	0.03	RNA processing and modification
K	237	6.80	Transcription
L	210	6.02	Replication, recombination and repair
B	2	0.06	Chromatin structure and dynamics
D	23	0.66	Cell cycle control, Cell division, chromosome partitioning
V	65	1.86	Defense mechanisms
T	100	2.87	Signal transduction mechanisms
M	92	2.64	Cell wall/membrane biogenesis
N	0	0.00	Cell motility
U	23	0.66	Intracellular trafficking and secretion
O	97	2.78	Posttranslational modification, protein turnover, chaperones
C	171	4.90	Energy production and conversion
G	128	3.67	Carbohydrate transport and metabolism
E	211	6.05	Amino acid transport and metabolism
F	69	1.98	Nucleotide transport and metabolism
H	100	2.87	Coenzyme transport and metabolism
I	187	5.36	Lipid transport and metabolism
P	191	5.48	Inorganic ion transport and metabolism
Q	84	2.41	Secondary metabolites biosynthesis, transport and catabolism
R	223	6.40	General function prediction only
S	1,116	32.00	Function unknown

### 3.5 Phylogenetic analysis of alkane hydroxylase gene and gene expression

**Figure 4:**
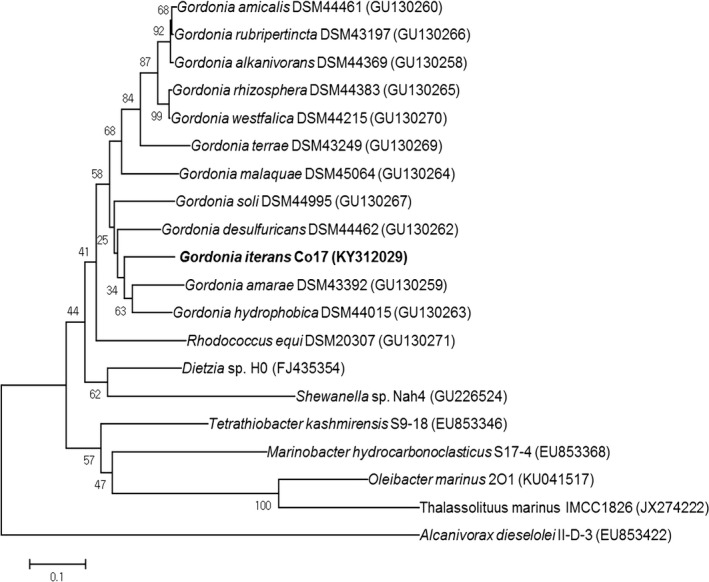
Phylogenetic relationship based on the complete sequences of *alk*B gene from *Gordonia* sp. Co17 and other bacteria was determined with the neighbor‐joining algorithm

The PCR was performed using the primers, alkBF and alkBR, and generates a product of 554 bp in length. The observed alkane hydroxylase (*alk*B) genes were confirmed based on the phylogenetic relationship with *Gordonia* species and other genera in Figure 4. The product *alk*B gene analyzed by NCBI blast showed the highest homology of 85% with *Gordonia hydrophobica* DSM44015 *alk*B gene (GU130263). The accession number of *alk*B gene of *G. iterans* Co17 sequence was obtained from the NCBI as KY312029.

**Figure 5:**
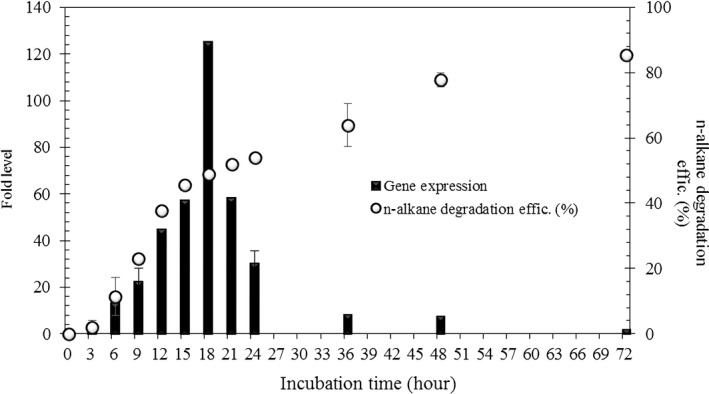
The relationship between *alk*B gene expression and *n*‐alkane removal efficiency. The alkB gene expression was normalized by 16 s rRNA reference gene. Error bars represent the standard deviation of duplicate samples

The relationship between *alk*B gene expression and *n*‐alkane *R*_E_ was monitored by qRT‐PCR analysis (Figure 5). The *alkB* gene was maximally expressed at 18 hours after the incubation with crude oil, and the expression level of 125 fold was up‐regulated. After 24 hr, the expression level of *alk*B gene gradually decreased. Meanwhile, the *R*_E_ was most rapidly increased (slope; 4.3) at 9 hr, and the slope of *R*_E_ was decline to 1.1 at 18 hr. After 48 hr, the slope of *R*_E_ decreased to 0.6, but it was confirmed that the residual amount *n*‐alkane was decreased continuously.

## 4 DISCUSSION

The strain Co17 exists in the oil phase on culture with crude oil (Supporting Information Figure S2, Olympus, Japan, X 1000), meanwhile did not observed emulsifying phenomena during culture period. In the year of 2003, reported that *Alcanivorax borkumensis*, the known to degrade crude oil in marine microorganisms, releases bio‐surfactant for glucose‐lipid and grown at the water/*n*‐hexadecane interphase (Golyshin, Martins Dos Santos, Kaiser, & Ferrer, 2003). But, newly isolated *Gordonia* sp. Co17 was grown at the oil phase and this suggests that it has a different bio‐surfactant for oil degradation pathway.

The *Gordonia* strains are known to degrade various kinds of oil. *Gordonia* sp. LE31 completely degraded the initial concentration of 1 g‐ diesel oil/L after 3 days (Lee, Kim, Kwon, & Park, 2005). *Gordonia* sp. JE‐1058 was removed 0.5 g of weathered Alaska North Slope crude oil by 82% after 28 days (Hong et al., 2010). *Gordonia* sp. strain 30A was degraded 10% (v/v) of *n*‐C_24_ (*n*‐tetracosane) nearly 50% at 60 hr (Matsui, Yamamoto, Shinzato, & Mitsuta, 2014). *Gordonia* CC‐JG39 decomposed 75% of the initial about 2,500 mg‐TPH (Total Petroleum Hydrocarbon)/kg after 23 days, and remained about 600 mg‐TPH/kg (Liu, Liou, Li, & Su, 2015). *Gordonia* sp. JG39 was reduced in freshwater with 500 mg‐ diesel oil/L, 94% of diesel degraded within 11 days (Chen, Whang, Pan, & Yang, 2017).

Specially, the *Gordonia* sp. Co17 has confirmed that it grow at low temperature (10°C) and high salinity (12%), and showed degradation efficiency under extreme condition. In the last 5 years, the average temperature of marine environment of the South Korea was 15–18°C and the salinity was 3.2% (KOSIS, KOrean Statistical Information Service). These results suggest that *Gordonia* sp. Co 17 was effective in bioremediation for contaminated marine areas including sediments. *Bacillus licheniformis* Y‐1 was isolated from heavy oil‐contaminated soil, and the strain could not utilize short‐chain alkane, but degraded long‐chain alkanes under high salinity conditions (Liu, Ju, Liu, & Wu, 2016). However, *Gordonia* sp. Co17 could degrade C_8_ to C_32_ range under 10% of NaCl concentration. Therefore, *Gordonia* sp. Co17 was expected to show good degradation efficiency when oil pollution in the marine occurs.

In similar with crude oil degradation, Al‐Wasify and Hamed (2014) have been reported that *Pseudomonas aeruginosa* removed 77.8% of Egyptian crude oil during 28 days at 22°C. The *R*_E_ values of *Bacillus subtilis* and *Acinetobacter lowffi* were 76.7% and 74.3%, respectively. The halotolerant strain *A. dieselolei* Qtet3 degraded a wide range of aliphatic hydrocarbons and long‐chain paraffin efficiently in saline conditions. *Alcanivorax dieselolei* exhibited 26.1% of hydrocarbon degradation efficiency at 2.5% salinity (Dastgheib, Amoozegar, Khajeh, & Ventosa, 2011). Cultured *Cellulosimicrobium cellulans* and *Acinetobacter baumannii* showed degradation efficiencies of 64.4% and 58.1%, respectively, after 10 days at an initial pH 7.5 and 32°C (Nkem, Halimoon, Yusoff, & Johari, 2016). Another TPH‐degrading bacteria *Dietzia cinnamea* P4 was utilized on average 75.5% of C_11_–C_36_ alkane, in BH media with Arabian light oil (von der Weid, Marques, Cunha, & Lippi, 2007). The *Pseudomonas* sp. JA5‐B45 degraded 13%–40% of crude oil with chemical surfactant (Igepal CO‐630) (Hamme & Ward, 2001).

In Dong paper (2012), the expression levels of two long‐chain fatty acid CoA ligases (*facl*1 and *facl*2) expressed by *Geobacillus thermodenitrificans* NG80‐2 were regulated when crude oil was cultured as a sole carbon source. These genes were related with degrade long‐chain alkanes with complex structure such as aromatic compounds. The *B. licheniformis* Y‐1 that isolated from heavy oil‐contaminated soil, and the strain could not utility short‐chain alkane, but also shown degradation rate with long‐chain alkane at high salinity condition (Liu et al., 2016). However, in this study *Gordonia* sp. Co17 was degraded in short chain and long chain alkane under 12% of salinity condition.

The expression of *alk*B in *Gordonia* sp. Co17 was analyzed by qRT‐PCR, and it appears that the *alk*B gene is secreted in the early stage of culture to degrade *n*‐alkane. Based on these results, it is considered that *Gordonia* sp. Co17 is shown to degrade *n*‐alkane by alkane hydrogenases other than *alk*B gene after 24 hr. Although the expression level of *alk*B gene is decreased, other *n*‐alkane degrading enzymes seem to be involved in the degradation efficiency.

We obtained crude oil degradation bacteria from oil‐contaminated sediment, especially the *Gordonia* sp. Co17 was shown highest efficiency 84.2%. Through the de novo whole genome sequencing, confirmed the alkane degrading related gene such as alkane‐1‐monooxygenase, alcohol dehydrogenase, and Baeyer–Villiger monooxygenase in *Gordonia* sp. Co17, and deposited to NCBI as the accession number CP027433. Specially, alkane‐1‐monooxygenase, *alk*B gene was the highest expressed 125‐fold at 18 hr, in during cultivation. Therefore, the *Gordonia* sp. Co17 might be an applied strain to degrade crude oil for bioremediation of crude oil‐contaminated soil as well as sediment in coastal area or deep sea.

## CONFLICT OF INTEREST

The authors declare that there are no conflict of interest. The complete genome sequence of *Gordonia iterans* Co17 has been deposited at Genebank under the accession number CP027433.

## AUTHORS CONTRIBUTION

Kim HS, Kim JS, and Lee SS designed experiments. Kim HS conducted experiments. Kim HS and Kim JS analyzed experiment results. DongKe analyzed WGS data. Kim HS, Kim JS, and DongKe wrote the manuscript.

## ETHICS STATEMENT

None required.

## Data Availability

All data are provided in full in the results section of this paper.
