# Supplementary figures and images for: Characteristics of crude oil‐degrading bacteria Gordonia iterans isolated from marine coastal in Taean sediment
Source: Microbiologyopen. 2018 Oct 19;8(6):e00754. doi: 10.1002/mbo3.754 (PMC6562140; doi:10.1002/mbo3.754)

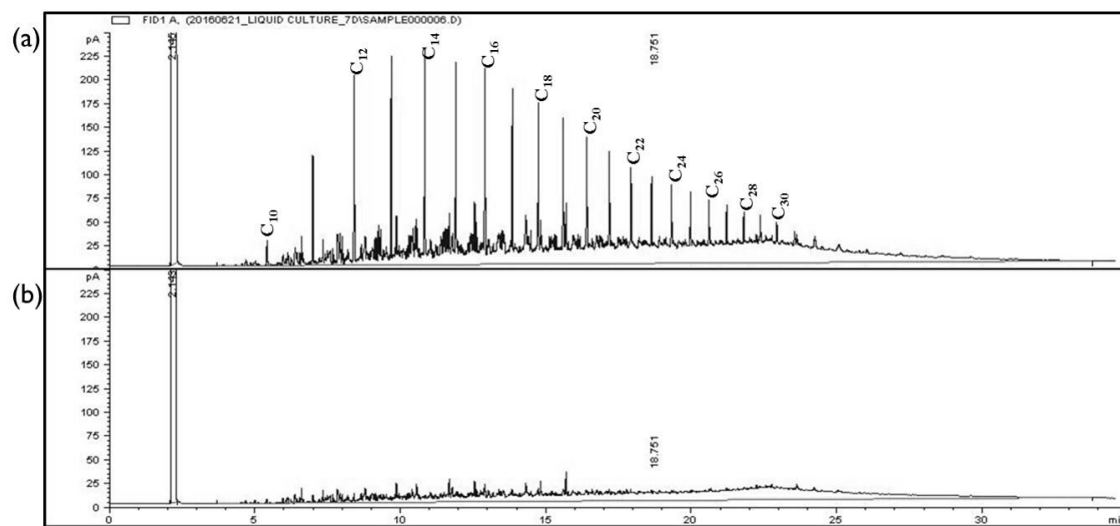

Supplementary Figure 1.

Supplement: Supplementary file 1 [file MBO3-8-e00754-s001.pdf]

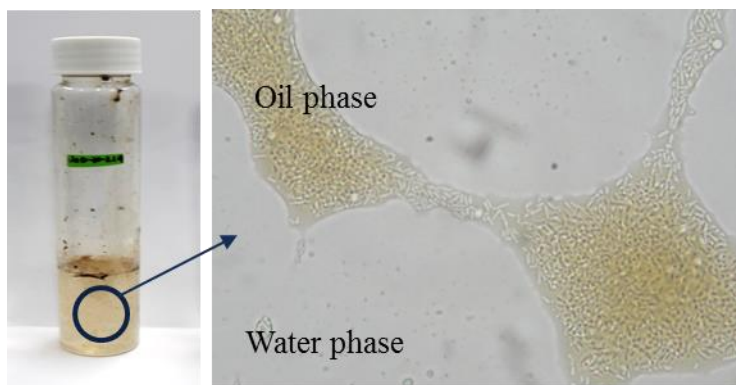

**Supplementary Figure 2.**

Supplement: Supplementary file 2 [file MBO3-8-e00754-s002.pdf]
