# Supplementary material for: Characteristics of crude oil‐degrading bacteria Gordonia iterans isolated from marine coastal in Taean sediment
Source: Microbiologyopen. 2018 Oct 19;8(6):e00754. doi: 10.1002/mbo3.754 (PMC6562140; doi:10.1002/mbo3.754)
Supplement: Supplementary file 3 [file MBO3-8-e00754-s003.docx]

Supplementary Table 1. *alk*B gene sequences obtained from NCBI.

| Strain | Sequence (5’ - 3’) | Accession number |
| --- | --- | --- |
| *Gordonia iterans* Co17 | GCCCCGCACGAGTTCGGGCACAAGAAGGTGTCCCTCGAGCGGTGGCTCTCCAAGGTGACGCTCGCTCAGACCGCCTACGGCCACTTCTACATCGAACACAACCGCGGCCACCACGTGCGGGTCGCGACTCCGGAGGATCCGGCGAGCTCGCGCTTTGGCGAGAACTTCTGGACCTTCTTGCCGCGCAGCGTGTTCGGCAGCCTCAAGTCCGCGTGGGAACTGGAGGCCAACCGCATGAAGCGACTCGAGCGGCCGGTCTTCCATCCCAGCAACGACGTCCTCAACGCCTGGGTGATGACCGTCGTCCTGTGGAGCGCGCTCACTGCGATCTTCGGCTGGGAGATCCTGCCCTTCATGGTGATTCAGGCGATCTATGGCTTCTCGCTGCTGGAGACCGTGAACTACCTGGAGCACTACGGCCTGCTGCGCCAGAAGAACAAGCGCGGACGTTACGAGCGGTGCACCCCGCAGCACAGCTGGAACTCCGACCACATCGCGACCAACATCTTCCTCTACCACCTGCAGCGGCACAGCGACCATCAGGGGGAACCCGA | KY312029 |
| *Gordonia amicalis*  DSM 44461 | GGAGTTGGGGCACAAGAAGGACGATCTCGAGCGCTGGCTGTCGAAGATCACGCTCGCGCAGACCTTCTACGGTCACTTCTACATCGAGCACAACCGTGGACATCACGTCCGCGTCGCCACCCCCGAGGACCCGGCGAGCTCGCGTTTCGGTGAGACCTTCTGGCGATTCCTGCCGCGGAGTGTGTGGGGCAGCCTGAAGTCGTCGATCGAGCTGGAGCGCAAGCGACTCGACCGTGTCGACAAGCCGTTCTGGCATCCGAGCAACGACGTCCTCAACGCCTGGGCCATGTCGATCGTGCTCTGGGGTGTGCTCGCGGCGGTGTTCGGGTGGCAGGTCCTGCCGTTCCTCGTCATCCAGGCCGTCTACGGGTTCTCGCTCCTCGAGACCGTCAACTACCTCGAGCACTATGGTCTGATGCGTCAGAAGACCAAGACCGGCCGCTATGAGCGCTGCACCCCGGAGCACAGCTG | GU130260 |
| *Gordonia rubripertincta*  DSM 43197 | GGACGATCTCGAGCGCTGGCTGTCGAAGATCACGCTCGCGCAGACCTTCTACGGTCACTTCTACATCGAGCACAACCGTGGGCATCACGTCCGCGTCGCCACCCCCGAGGACCCGGCGAGCTCGCGTTTCGGTGAGACCTTCTGGCGATTCCTGCCGCGGAGTGTGTGGGGCAGCCTGAAGTCGTCGATCGAGCTGGAGCGCAAGCGACTCGACCGTGTCGACAAGCCGTTCTGGCATCCGAGCAACGACGTCCTCAACGCCTGGGCCATGTCGATCGTGCTGTGGGGTGTGCTCGCGGCGGTGTTCGGGTGGCAGGTCCTGCCGTTCCTCGTCATCCAGGCCGTCTACGGGTTCTCGCTCCTCGAGACCGTCAACTACCTCGAGCACTACGGTCTGATGCGTCAGAAGACCAAGACCGGACGCTACGAGCGCTGCACCCCGGAGCACAGCTG | GU130266 |
| *Gordonia alkanivorans*  DSM 44369 | GGACGATCTCGAGCGCTGGCTGTCGAAGATCACGCTCGCGCAGACCTTCTACGGTCACTTCTACATCGAGCACAACCGTGGGCATCACGTCCGCGTCGCCACCCCCGAGGACCCGGCGAGCTCGCGTTTCGGTGAGACCTTCTGGCGATTCCTGCCGCGGAGTGTGTGGGGCAGCCTGAAGTCGTCGATCGAGCTGGAGCGCAAGCGCCTCGACCGTGTGGGCAAGCCGTTCTGGCATCCGAGCAACGACGTCCTCAACGCCTGGGCCATGTCGATCGTGCTGTGGGGTGTGCTCGCGGCGGTGTTCGGGTGGCAGGTCCTGCCGTTCCTCGTCATCCAGGCCGTCTACGGGTTCTCGCTCCTCGAGACCGTCAACTACCTCGAGCACTACGGTCTGATGCGTCAGAAGACCAAGACCGGCCGCTACGAGCGCTGCACCCCGGAGCACAGCTG | GU130258 |
| *Gordonia rhizosphera*  DSM 44383 | ACGATCTCGAGCGCTGGCTGTCGAAGATCACGCTCGCGCAGACCTTCTACGGGCACTTCTACATCGAGCACAACCGCGGGCATCACGTCCGCGTCGCCACCCCCGAGGACCCGGCGAGCTCGCGCTTCGGTGAGACCTTCTGGCGATTCCTGCCGCGCAGCGTGTGGGGCAGCCTGAAGTCGTCGATCGAGTTGGAGCGCAAGCGCCTCGACCGTGCGGGCAAGCCGTTCTGGCACCCGAGCAACGACGTCCTCAACGCGTGGGCGATGTCGATCGCGTTGTGGGGTGTGCTCGCCGCCGTGTTCGGCTGGCAGGTCCTGCCGTTCCTCGTCATCCAGGCCGTCTACGGGTTCTCGCTGCTCGAGACCGTCAACTACCTCGAGCACTACGGCCTGATGCGCCAGAAGACCAAGACCGGACGCTACGAGCGCTGCACTCCGGAGCACAGCTGGAACTCCGATCACATCTGCACGAACATCT | GU130265 |
| *Gordonia westfalica*  DSM 44215 | CGATCTCGAGCGCTGGCTCTCGAAGATCACGCTCGCGCAGACCTTCTACGGGCACTTCTACATCGAGCACAACCGCGGGCATCACGTCCGCGTCGCCACCCCCGAGGACCCGGCGAGCTCGCGCTTCGGTGAGACCTTCTGGCGATTCCTGCCGCGCAGCGTGTGGGGCAGCCTGAAGTCGTCGATCGAGTTGGAGCGCAAGCGCCTCGACCGTGCGGGCAAGCCGTTCTGGCACCCGAGCAACGACGTCCTCAACGCGTGGGCGATGTCGATCGCGTTGTGGGGTGTGCTCGCCGCCGTGTTCGGCTGGCAGGTCCTGCCGTTCCTCGTCATCCAGGCCGTCTACGGGTTCTCGCTGCTCGAGACCGTCAACTACCTCGAGCACTACGGCCTGATGCGCCAGAAGACCAAGACCGGACGCTACGAGCGCTGCACCCCGGAGCACAGCTGGAACTCCGATCACATCTGCACGAACATCTTCCTGTATCACCTTCA | GU130270 |
| *Gordonia terrae*  DSM 43249S | CACAAGAAAGACGACCTCGAGCGCTGGCTGTCGAAGATCACGCTCGCCCAGACCTTTTACGGTCACTTCTTCATCGAGCACAACCGCGGCCACCACGTGCGGGTCGCGACGCCCGAGGACCCGGCCAGCTCGCGGTTCGGAGAATCGTTCTGGCGCTTCCTTCCCCGCAGCGTCTGGGGAAGTCTGAAGTCCTCGGTCGAACTCGAACGCAAGCGACTCGAGCGTGCGGACAAGCCGTTCTGGCACCCGAGCAACGACGTCCTCAACGCCTGGGCGATGTCGATCGTGTTGTGGGGTGCGCTCACGGCGATCTTCGGCTGGGAGATCCTGCCGTTCCTGGTGATCCAGGCCGTGTACGGCTTCTCGCTGCTGGAGACCGTGAACTATCTCGAGCACTACGGTCTGCTCCGCCAGAAGACCGCGACCGGTCGCTACGAACGATGCACTCCGGAGCACAGCTG | GU130269 |
| *Gordonia malaquae*  DSM 45064 | TCGAGCGCTGGCTGTCGAAGATCACTCTCGCGCAGACGTTCTACGGTCACTTCTTCATCGAGCACAATCGTGGTCATCACGTCCGCGTCGCCACCCCCGAGGATCCGGCGAGCTCGCGCTTCGGTGAGACCTTCTGGTCGTTCCTGCCGCGCAGTGTCTACGGCAGTCTGAAGTCGGCATGGGAACTCGAGGCCAACCGCCTCAAGAAGATCGACAAGCCGGTGTTCCATCGGAGCAACGACGTTCTCAACGCGTGGGTCATGTCGATCGTCCTGTGGGGTGTGCTCGCAGCGGTCTTCGGCTGGCAGATCCTGCCCTTCATGGTCCTGCAGGCCGTCTACGGTTTCTCGCTGCTGGAGACCGTCAACTATCTCGAGCACTACGGCCTCGTCCGGCAGAAGAACGCCCGCGGTCGTTACGAGCGCTGCACCCCGTCACACAGCTGGAACAGTGACCACATCTGCAC | GU130264 |
| *Gordonia soli*  DSM 44995 | CCATGAGTTGGGGCACAAGAAGGACTCGCTCGAGCGCTGGCTCTCCAAGATCACCCTGGCGCAGACGTTCTACGGCCACTTTTACATCGAGCACAACCGCGGGCATCACGTGCGCGTGGCGACTCCCGAGGATCCCGCCAGCTCACGCATGGGCGAGAACTTCTGGACCTTCCTGCCCCGCAGCGTGGTCGGCAGCCTGCGCTCGTCGTGGGAACTCGAGGCCAAGCGGATGCAGCGTCTCGAGAAGCCGGTATGGCACCCGAGCAACGACGTCCTCAACGCCTGGGCGATGTCGGTGGTGCTCTGGGGCGCTCTCGCAGCCGTCTTCGGGTGGCAGGTGCTGCCGTTCCTGGTGATCCAGGCGATCTATGGCTTCACACTGCTGGAGACGGTCAACTACCTCGAGCACTACGGACTCCTACGGCAGACCACCGCGCGCGGCCGGTACGAGCGCTGCACTCCGCAGCACAGC | GU130267 |
| *Gordonia desulfuricans*  DSM 44462 | CTACGGCCACTTCTACATCGAGCACAACCGCGGCCACCACGTGCGCGTCGCGACCCCGGAGGACCCCGCGAGCTCACGTTTCGGTGAGAGCTTCTGGACCTTCCTGCCGCGCAGCGTGTTCGGCAGCCTCCGCTCGGCCTGGGAGCTGGAGGCCAAGCGGATGGAACGCCTCGACCGGCCGGTGTGGCACCCCAGCAACGACGTCGTCAACGCCTGGGCGATGACCATCGTGCTGTGGGGGGCGCTGGCGGCGATCTTCGGCTGGCAGGTGCTGCCGTTCGCGCTGATCCAGGCAGTCTACGGGTTCTCCCTGCTGGAATCGGTGAACTACCTCGAGCACTACGGGCTGCTGCGGCAGAAGACCGCGAGCGGGCGCTACGAACGGTGCGCCCCGCGACACAGCTGGAACTCCGACCACATCTGCACCAACATCTTCCTCTATCACCTGCA | GU130262 |
| *Gordonia amarae*  DSM 43392 | GTGACGTTGGCGCAGACCTGCTACGGACACTTCTACATCGAGCACAATCGCGGCCACCACGTCCGCGTCGCGACCCCGGAGGACCCGGCCACCTCCCGATTCGGTGAGAGCTTCTGGCGATTCCTGCCGCGCAGTGTGTTCGGCAGCCTCAAGTCCGCGTGGGAGCTGGAGGCGACCCGTCTCAAGCGTCTCGGGCGTCCGGTCCTGCATCCGAGCAACGACGTCCTGAATGCCTGGGCGATGTCGATCGTGCTGTGGGGCGTGCTGACCGCGATCTTCGGATGGCAGATCCTGCCCTTCGCGGCACTGCAGGCCGTCTACGGTTTCTCGCTGCTCGAATCGGTGAACTTCCTGGAACACTACGGACTGCTGCGGCAGAAGAACGAGCGCGGCCGCTACGAGCGCTGCACGCCGGAACACAGCTGGAACAGCGACCACATCTGCACCAACATCTTCCTGTACCACCTTCAGC | GU130259 |
| *Gordonia hydrophobica*  DSM 44015 | TCACCATGGCGCAGACCTTCTACGGCCACTTCTACATCGAGCACAACCGCGGCCACCACGTCCGCGTGGCGACGCCGGAGGACCCCGCGAGCTCCCGGTTCGGCGAGAACTTCTGGACCTTCTTGCCGCGCAGCGTGTTCGGCAGCCTGAAGTCCGCGTGGGAGCTCGAGGCCACACGACTGCGGCGCGCGGACAAGCCCGTCTTTCATCCGAGCAACGACGTCCTGAACGCCTGGGCCATGTCGATCGTCCTGTGGGGCGGACTGACCGCGATCTTCGGGTGGCAGATCCTGCCGTTCGTCGCCATCCAGGCCGTCTACGGATTCTCCCTGCTGGAGACCGTCAACTACTTGGAGCACTACGGACTGCTGCGGCAGAAGAACGAGCGCGGCCGCTACGAGCGCTGCACCCCGCAGCACTCGTGGAACAGCGACCACATCTGCACCAACATCTTCCTCTACCACCTGCAGC | GU130263 |
| *Rhodococcus equi*  DSM 20307 | GTCGAGATCACGCTCGCGCAGTCGTTCTACGGCCACTTCTATATCGAGCACAACCGCGGCCACCACGTGCGGGTCGCGACCCCCGAGGACCCCGCGAGTTCACGCTTCGGGGAGAGCTTCTGGGCATTCCTCCCGCGCAGCGTGTGGGGCAGTCTGCGGTCGTCCTGGCGCCTGGAGAAGGCGCGCCTGGAGCGGCTCGGTAAGGGGCCGTGGACAATTCGGAACGACGTGCTGAACGCGTGGCTGATGTCGGTGGTGTTGTTCGGCGCGATGATCGCGATCTTCGGCTGGGAAGTGGCGCCGTTCCTGTTGCTGCAGGCCGTGTTCGGGTTCTCCCTGCTGGAGACCGTCAACTATCTCGAGCACTACGGGTTGCTGCGGCAGCGCACCGAGAGTGGGCGTTACGAACGGTGCACGCCCGCTCACAGCTGGAACAGCGATCACATCTGCACCAACATCTTCCTGTACCACCTGCA | GU130271 |
| *Dietzia* sp. H0 | GATCGCCGGCAGCGAGAAGTGGCTGTCCAAGGTCGCCCTGGCCACCACCGGCTACGGACACTTCTTCATCGAGCACAACCGCGGACACCACGCGCGTGTCGCCACTCCGGAGGATCCGGCCAGCTCGCGCTTCGGTGAGTCGTTCTGGGCCTTCCTCCCGCGCAGCGTGTTCGGGTCGCTGACGTCGGCCTGGCACCTGGAGTCCGAGCGGCTCCGGCGGCTGGGCAAGAGCCCGTGGACCCTGCGCAACGACAACCTCAACGCCTGGCTCATGACGGTCGTGCTCTTCGGCGGACTCATCGCCGTGTTCGGCTGGGAGATCGCGCCGTGGCTCATCGTGCAGGCCGTCTTCGGCTTCTCGCTGCTGGAGGTCGTCAACTACCTCGAGCACTACGGCCTGCTCCGCCAGAAGACCTCCGCCGGCCGCTATCAGCGCTGCCGCCCGGAGCACTCGTGGAACTCCGACCACCTGGTGACCAACATCTTCCTCTACCACCTG | FJ435354 |
| *Shewanella* sp. Nah4 | CACAACCACGGCCACCATCTGCGCGTCGCCACCCCCGAGGATCCGGCGTCGGCGAAATTCGGTGAATCGTTCTGGAAGTTTCTGCCACGCACGATGATCCACGGTCTGCATTCGGCCTGGGATCTCGAAACCCGTCGACTCGCGCGGTCGGGCTCGTCGCCGTGGACGCTGCGCAACAACCTTTTCAACGCGGCCGCGATGAGCATCGTTCTGTTCGGGGCACTGATCTCAGTGTTCGGTTGGATCGTTTTGCCGTACCTACTGATTCAGGCCGGCATCGCCATCGTCCTGTACGAGGCGGC | GU226524 |
| *Tetrathiobacter kashmirensis*  S9-18 | TATACGTCCCATGAGCTTGGTCACAAAACCAACTCGCTGGAGCGCTGGCTGGCCAAGCTGACGCTGGGTCCAGTGGCCTATGGGCACTTCTTCATCGAGCACAACAAGGGCCACCACAAGAACGTGGCGACGCCGGAAGATCCGGCCAGCTCGCGGATGGGCGAAACCTTCTGGCAGTTCCTGCCGCGCACGATGAGTGGCAGCCTGCGGTCCGCATGGGAGATCGAGCGCAACCGTCTTGAGCGCAACGGCCAGAGCGTGTGGAGCATTCATAACGAGAACCTTCAGGCTTGGGCGATGACGGTGCTGCTGTTCGGTGGCCTGACCGCTTGGCTAGGCTGGCCCGCGCTGGTCTTCCTCGGCGCCCAGGCGTTCTACGGCGCCTCGCTGCTGGAGGTGGTCAACTATCTGGAGCATTACGGCCTGTGCCGTCAGAAGCTGCCGAGCGGCCGTTATGAGCGCTGCGCGCCGCGCCATTCCTGGAACAGCAACCACATCGTGACCAACCTGTTCCTGTATCAGCTGCAGCGGCACTCGGATCACCATGCA | EU853346 |
| *Marinobacter hydrocarbonoclasticus*  S17-4 | AATACTGCACATGAGCTTGGTCACAAGTCCAATAAACTGAACAAACTGATGGCCATGGCCGCCTTGGCCCCCACCGCCTACACCCACTTTGTGGTGGAACATAATTTTGGTCATCACAAGCGGGTAGCCACACCGGAGGACCCCGCCAGCAGCCGAATGGGCGAGAGTTTCTGGAAGTTCCTGCCCCGAACCGTGTTCGGCGGCATCAAGTCGTCGATCAAGATCGAAAAGGCACGCCTGGAACGCAAGGGTAAGAGCTTCTGGAGTCTTGATAACGAACTGCTACAGGGTTGGGCCATGACGGCCGGGTTCTTTGGCGCCACGACGCTGGTCTGCGGCCCGAGGGCGGTGCCGTTCCTGGCGGCACAGGCGGTTTATGGCGCCAGTCTGTTGGAGAGTGTGAATTACATCGAGCACTATGGTCTGTTGCGGCAGAAGGACAAGAATGGCAAGTATGAGCGCACGAAGCCGGAACACAGCTGGAACAGTAATCACATTGTGACCAACCTGTTCCTGTATCAGTTGCAACGGCATTCAGACCACATGCA | EU853368 |
| *Oleibacter marinus*  2O1 | CGGTGGTCTGCTGCTGGCTCTGGTTACATATGCAGGTTTTAAAGTTGAGCATATCCGTGGTCACCATGTGCATGTATCAACGCCTGAAGACGCGTCTTCATCACGCTACAACCAGACTCTGTATCAGTTCCTGCCGCACGCGTATTTCCACAATTTCATGAATGCGTGGAAACTCGAAGCGAAAAAACTGAAGCGCAAAAATCTGCCAGCGCTGCACTGGAAAAATGAACTGATCTGGTGGTACGCCATTTCAGCGGCAACCATGATCGGTTTCGGCCTGGTATGGGGCTGGATGGGTGCCCTGTTCTTCATCGCGCAGAGCTTCTTCGCCTTCACCCTGCTGGAAATCGTTAACTACATTGAGCACTACGGTCTGCACCGTCGTAAGCTGGAAAACGGCCGCTACGAGCGCACGACCCCAGCGCACTCATGGAACAGTAACTTCCTGCTGACCAACCTGTTCCTGGTACAACTGCAGCGGCACTCCG | KU041517 |
| *Thalassolituus marinus*  IMCC1826 | AAGACACCAGAACAGAGCAATGGAGTGGTGGCTTATTACTGGCGACCGTGTGTTATTCCGGTTTTAAGGTTGAGCATATTCGCGGCCATCATGTGCACGTTTCAACGCCCGAGGACGCTTCCTCGTCGCGCTACGGCCAATCGCTGTACCAGTTTCTGCCACATGCGATCGTGCACAACACCGCCAATGCCTGGAAGCTGGAAGCGCAGCGTCTGCGCAAAGCCGGACACTCACCGTTAAGCTGGCACAATGAGCTGATCTGGTGGAATGTTATCAGTATTGCCTTAGCGGCCATGTTTACTCTGGCCTGGAGTTGGATGGGGCTGGTGTACTTCCTGGGCCAGAGTCTGGTCGCCATTACTCTGCTGGAAATCATTAACTATGTTGAGCACTACGGCTTACACCGTCGCAAACTGGATAACGGCCGTTATGAGCGCACAACCATTGAGCACTCCTGGAACAGCAATTACCTGCTGACCAATCTGTTCCTG | JX274222 |
| *Alcanivorax dieselolei*  II-D-3 | TCCGTAGTGTTCGATGTAATTGGCGGAGGTCAACTGGAACGCGCCCCAGAAAGCGGTGCCGAGGATGTACGGCAGAATGCCGATGCCGAACAACGCCAGCACGACTCCCCAGGCAATGGCCGTGATGATCGCCGGCTGAATGATTTCGTTGTCCAGGGACCACACCGATTTGCCCCGTGATTCCAGGCGCTCCTGCTCCAGCTTCCAGGCCCGGCGGGCGGCACCGGGGATCTCCCGCAGTACGAATTTCCAGATGCTTTCGCCCATGCGCGAGGATGCCGGGTCCTCCGGCGTGGCCACGTCCCGGTGATGACCCTTGTTGTGCTCAATGAAAAAGTGGCCGTAGGCGCAGGGCGCCAGCACGAACTTGGCCAGCCAGCGTTCGCCCTTGCCTTTCTTGTGGCCCAGTTCATGTCCGGTGTT | EU853422 |

Supplementary Table 2. Total 144 isolates from contaminated site. The crude oil degradation efficiency detected on 7days. *None; Strain does not exhibit crude oil degradation efficiency.

| Strain | Efficiency (%) | Strain | Efficiency (%) | Strain | Efficiency (%) |
| --- | --- | --- | --- | --- | --- |
| Co17 | 84.23 | PHEN7 | 49.85 | A29 | 15.40 |
| Py 2-4 | 80.87 | L9 | 49.81 | Arti 3 | 15.20 |
| B-0-12 | 76.59 | Po9 | 49.75 | L20-2 | 14.90 |
| Po7 | 75.88 | L4-2 | 47.25 | L20-1 | 13.80 |
| S. PHEN6 | 72.31 | 20D-30-3 | 47.28 | L25 | 13.20 |
| Wonp3 | 69.80 | UL1 | 47.20 | L8 | 13.10 |
| PHEN12 | 69.75 | 20S-30-2 | 47.20 | L30 | 12.50 |
| LNB035-2 | 69.23 | Po5 | 47.20 | L17 | 12.20 |
| Po6 | 69.21 | UL3 | 47.20 | L23-1 | 10.80 |
| F3 | 69.20 | PHEN8 | 46.80 | L29-1 | 10.80 |
| 9004-010 | 69.20 | UL11 | 46.80 | L15-1 | 10.70 |
| UL13 | 69.15 | UL15 | 46.70 | A18 | 10.50 |
| F8 | 69.15 | UL9 | 45.80 | A28 | 10.50 |
| PHEN13 | 69.10 | Po10 | 43.50 | L19 | 10.30 |
| TS13 | 69.00 | UL10 | 42.10 | A15 | 9.90 |
| 20S-25-12 | 68.87 | A12 | 41.60 | A30 | 9.90 |
| 20S-25-5 | 68.74 | TS 16 | 41.20 | A20 | 9.40 |
| 20S-25-11 | 68.65 | UL8 | 40.90 | A39-1 | 9.00 |
| LNB024-2 | 68.54 | UL4 | 36.70 | A26-1 | 8.90 |
| 20S-25-10 | 68.44 | Oilp1 | 35.70 | A3 | 8.90 |
| 20S-25-14 | 68.32 | A9 | 35.20 | L1 | 8.30 |
| 20D-30-2 | 67.89 | UL7 | 35.10 | S31 | 8.20 |
| UL6 | 67.75 | UL5 | 33.70 | A5 | 7.90 |
| Wonp9 | 67.15 | Po11 | 33.20 | A2 | 7.40 |
| 9004-035 | 67.11 | UL12 | 33.10 | L10 | 6.70 |
| 20D-30-4 | 66.80 | A16 | 32.80 | L23 | 6.40 |
| 20S-25-4 | 66.75 | UI-2 | 32.80 | A8 | 6.20 |
| 20S-25-1 | 66.12 | UL2 | 32.80 | L25-1 | 5.80 |
| 20S-25-8 | 65.87 | L16 | 32.50 | Arti 2 | 5.60 |
| 20S-25-3 | 65.77 | Po12 | 31.20 | A25 | 4.50 |
| 20S-25-13 | 65.43 | Po3 | 31.20 | A27 | 3.40 |
| 20S-25-2 | 64.74 | L26-1 | 29.20 | S29 | 3.4 |
| 20S-25-15 | 64.65 | Po2 | 28.80 | A4 | 1.8 |
| 20S-25-16 | 64.15 | Po4 | 28.80 | A24 | 0.7 |
| NB31 | 63.75 | L4-1 | 25.90 | A7 | 0.4 |
| PHEN4 | 62.90 | A38 | 25.80 | CF1 | None |
| 20S-25-6 | 61.85 | Po1 | 25.80 | A31 | None |
| 20S-25-9 | 59.87 | A6 | 25.40 | A23 | None |
| 20D-30-5 | 59.75 | L24 | 23.60 | A37 | None |
| F4 | 58.75 | A35-1-1 | 22.80 | Fluo1 | None |
| Po8 | 58.70 | A36 | 21.90 | A33 | None |
| L29-2 | 58.65 | A19 | 21.20 | L11-1 | None |
| 9004-034 | 56.55 | A1 | 21.00 | L16-1 | None |
| TS11 | 55.10 | Arti 1 | 18.20 | L26 | None |
| 20S-25-7 | 54.90 | L21 | 17.30 | A36-1 | None |
| PHEN16 | 53.28 | A30-1 | 15.80 | L11 | None |
| TS 14 | 50.15 | A39 | 15.47 | A40-1 | None |
| A34 | 50.08 | A26 | 15.40 | L15 | None |

Supplementary Table 3. Classification and general feature of *Gordonia iterans* Co17.

| Property | Term |
| --- | --- |
| Classification | Domain *Bacteria* Phylum *Actinobacteria* Class *Actinobacteria*  Order *Actinomycetales* Family *Gordoniaceae* Genus *Gordonia* |
| Gram stain | Positive |
| Cell shape | Rod |
| Motility | Non-motile |
| Sporulation | Not reported |
| Optimum temperature | 28 °C |
| pH range; Optimum | 6.0-11.0; 7.0 |
| Salinity | 0-12 % |
| Oxygen requirement | Aerobic |
| Latitude and Longitude | 36°47'16"N 126°08'37"E |
| Geological Location | South Korea; Tae-an coastal |
| Collection data | March 2008 |
| Collection Site | Ocean; Marine sediment; Contaminated sediment |
| Estimated size | 4,006,502 |
| Sequence method | PacBio RSII, Illumina platform |
| Annotation source | GenBank |
| Finishing strategy | Complete; 161× coverage, 1 contig |
| Assembly method | HGAP3 |
| Genome coverage | 161× |
| Locus Tag | C6V83 |
| Genbank ID | CP027433 |
| BIOPROJECT | PRJNA436863 |
| Sequencing technology | PacBio, Illumina |
